# First detection of Jingmen tick virus in Corsica with a new generic RTqPCR system

**DOI:** 10.1038/s44298-024-00053-1

**Published:** 2024-09-30

**Authors:** Vincent Cicculli, Agathe M. G. Colmant, Géraldine Piorkowski, Rayane Amaral, Apolline Maitre, Dorine Decarreaux, Laurence Thirion, Gregory Moureau, Alessandra Falchi, Xavier de Lamballerie, Remi N. Charrel, Nazli Ayhan

**Affiliations:** 1 Unite des Virus Emergents, (UVE: Aix-Marseille Univ, Universita di Corsica, IRD 190, Inserm 1207, IRBA), Marseille, France; 2 ANSES, INRAE, Ecole Nationale Vétérinaire d’Alfort, UMR BIPAR, Laboratoire de Santé Animale, Maisons-Alfort, France; 3 INRAE, UR 0045 Laboratoire de Recherches Sur Le Développement de L’Elevage (SELMET-LRDE), Corte, France; 4 Centre National de Référence des Arbovirus, Inserm-IRBA, Marseille, France

**Keywords:** Virology, Viral epidemiology

## Abstract

Jingmen tick virus (JMTV) is a recently discovered segmented RNA virus, closely related to flaviviruses. It was identified for the first time in 2014, in China and subsequently in Brazil. Following this discovery, JMTV-related sequences have been identified in arthropods, vertebrates (including humans), plants, fungus, and environmental samples from Asia, America, Africa, Europe, and Oceania. Several studies suggest an association between these segmented flavi-like viruses, termed jingmenviruses, and febrile illness in humans. The development of rapid diagnostic assays for these viruses is therefore crucial to be prepared for a potential epidemic, for the early detection of these viruses *via* vector surveillance or hospital diagnosis. In this study, we designed a RT-qPCR assay to detect tick-associated jingmenviruses, validated it and tested its range and limit of detection with six tick-associated jingmenviruses using in vitro transcripts. Then, we screened ticks collected in Corsica (France) from different livestock species, in order to determine the distribution of these viruses on the island. In total, 6269 ticks from eight species were collected from 763 cattle, 538 horses, 106 sheep, and 218 wild boars and grouped in 1715 pools. We report the first detection of JMTV in Corsica, in *Rhipicephalus bursa*, *Hyalomma marginatum* and *R. sanguineus* ticks collected from cattle and sheep. The highest prevalence was found in the *Rhipicephalus* genus. The complete genome of a Corsican JMTV was obtained from a pool of *Rhipicephalus bursa* ticks and shares between 94.7% and 95.1% nucleotide identity with a JMTV sequence corresponding to a human patient in Kosovo and groups phylogenetically with European JMTV strains. These results show that a Mediterranean island such as Corsica could act as a sentinel zone for future epidemics.

## Introduction

Jingmen tick virus (JMTV) is a segmented RNA virus that was identified for the first time in 2014 in ticks from the Jingmen region of the Hubei province in China and simultaneously from the Mogiana region of Brazil^1,2^. The genome comprises four segments of positive-sense single-stranded RNA. Segments 1 and 3 encode non-structural proteins, genetically and functionally close to the non-structural proteins NS3 and NS5 of members of the *Ortholavivirus* genus in the *Flaviviridae* family. Segments 2 and 4 encode putative structural proteins, which are not as closely related to flavivirus structural proteins as the non-structural proteins are ref. ^2^.

Following this discovery, JMTV RNA was detected in arthropods (including ticks within the *Rhipicephalus, Haemaphysalis, Ixodes, Dermacentor, Amblyomma, Hyalomma* genera), reptiles and mammals (including cattle and humans) from all continents, alongside other related segmented flavi-like virus sequences termed jingmenviruses^1–7^. The jingmenvirus sequences group phylogenetically into two clades: tick-associated jingmenviruses (also found in vertebrates), and sequences detected from insects (type species: Guaico Culex virus, from *Culex* mosquitoes), crustaceans, plants and fungi^8^.

Two tick-associated jingmenviruses have been found in Europe: JMTV and Alongshan virus (ALSV). JMTV RNA has been detected in humans from Kosovo, in field-collected ticks from Türkiye and Romania, and in an *Aedes albopictus* mosquito laboratory colony in Italy, while ALSV RNA was found in ticks from Finland, France, Germany and Switzerland^3,9–16^. Concurrently, these are the only two jingmenviruses that have been found in humans: JMTV and ALSV-derived RNA and antibodies were detected in patients with tick bites and febrile illness in China and Kosovo^5,9,17^.

The tick-associated jingmenvirus clade also includes Yanggou tick virus (YGTV) found in ticks from China and Russia, Takachi virus (TAKV) in ticks from Japan, Pteropus lylei jingmenvirus (PLJV) in bats from Cambodia, a partial genome from mice from the United States of America (Peromyscus leucopus jingmenvirus) and two divergent full genome sequences from cattle feces and soil samples from China (Guangdong jingmen-like virus, GJLV and Hainan jingmen-like virus)^3,18–22^.

To date, it is still unknown whether the emerging potential of jingmenviruses warrants their integration in surveillance campaign, and whether they might play an indirect role in the ecology of arboviruses. Despite these gaps in knowledge, no analytically validated molecular assay is available for tick-associated jingmenviruses at this time. Such an assay could help elucidate the vector competence of different tick species and the transmission cycle of jingmenviruses, therefore facilitating the prediction of virus introduction into new territories. The aim of this study was to design and validate a real-time RT-qPCR assay for the detection of tick-associated jingmenviruses, and to generate epidemiological data in Corsica, a French Merditerranean island, by screening ticks collected from farmed and wild animals on the island. We chose Corsica because: (i) many genera of ticks, such as *Ixodes, Hyalomma, Dermacentor, Haemaphysalis, Rhipicephalus,* and *Amblyomma*, are present^23–25^ and (ii) the circulation of zoonotic diseases in Corsica is facilitated by the widespread practice of mixed livestock farming, the presence of avian migration corridors, and strong interactions between livestock, wildlife and human populations^23,26^.

## Results

### Molecular detection system selection

In this study, we designed two systems to detect tick-associated jingmenviruses genomic segments 1 or 2, named gTJ-seg1 and gTJ-seg2. In order to ensure that these were functional, a plasmid containing the region targeted by both systems was diluted from 100 pg/µL to 0.1 pg/µL and used as template in qPCR assays with gTJ-seg1 or gTJ-seg2. Both systems were indeed functional, gTJ-seg2 was more sensitive than gTJ-seg1 by 3 to 7 Ct (Table 1) and was therefore selected to be used for large-scale screening of all tick pools.

**Table 1:** Cycle thresholds (Ct) obtained with gTJ-seg1 and gTJ-seg2 on serial dilutions of plasmids containing both systems’ targets in JMTV

	gTJ-seg1		gTJ-seg2	
Plasmid concentration	Replicate 1	Replicate 2	Replicate 1	Replicate 2
100 pg/µL	22.9	22.9	17.9	17.6
10 pg/µL	25.8	25.8	22.8	22.8
1 pg/µL	31.7	32.2	24.3	24.5
0.1 pg/µL	35.1	35.3	27.8	27.8

### Range and limit of detection of the gTJ-seg2 system

Tripling dilutions of in vitro transcribed (IVT) RNAs corresponding to increasingly divergent jingmenviruses in the tick-associated clade were used in a RT-qPCR to determine the range of virus sequences detected by gTJ-seg2. A RT-qPCR system detecting actin was used alongside as a positive control for all IVTs.

**Fig. 1:**
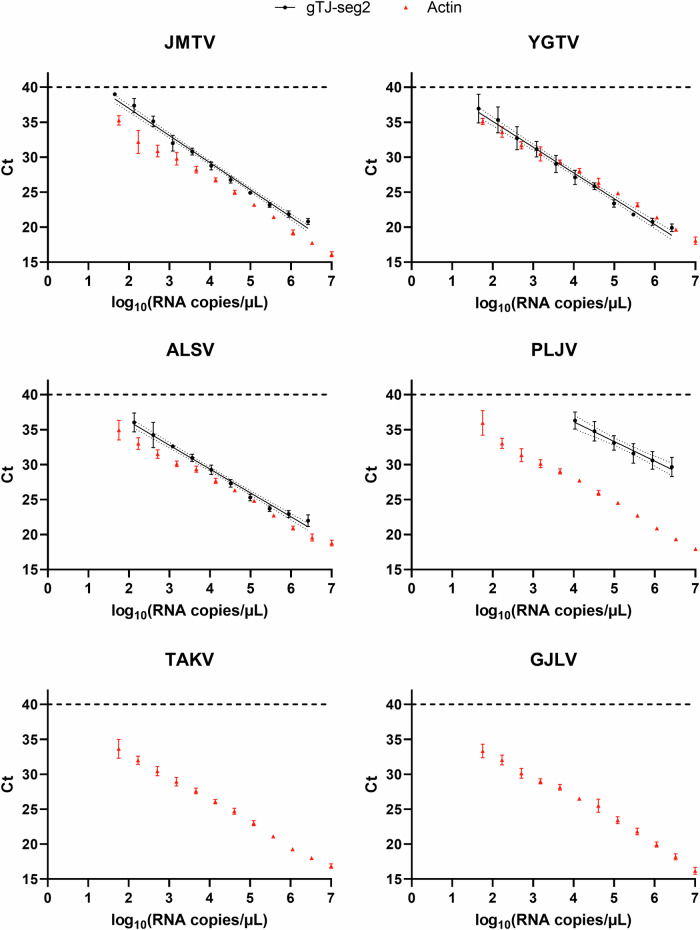
Range and limit of detection of gTJ-seg2. IVTs of six increasingly divergent tick-associated jingmenvirus sequences were serially diluted in multiple replicates and used in RT-qPCR with gTJ-seg2 (black discs, *n* = 4) and an actin system (red triangles, *n* = 8). These graphs represent only the Ct < 40 (cycle threshold) obtained, as a function of the quantity of template used for both systems. All tested dilutions (up to 10^10^ RNA copies /µL) were negative for TAKV and GJLV using gTJ-seg2. Dashed line: limit of detection of the RT-qPCR assays: Ct = 40; straight line: linear regression of gTJ-seg2 data; dotted lines: 95% confidence intervals, both estimated using GraphPad Prism 9.4.1; error bars represent the standard deviation.

The lowest limits of detection were obtained for YGTV, JMTV and ALSV (140 RNA copies / µL, 245 RNA copies/µL and 298 RNA copies/µL respectively) (Fig. 1 and Supplementary Table 1). PLJV was able to be detected at high IVT concentrations (limit of detection: 2.8 × 10^4^ RNA copies/µL) while TAKV, GJLV remained negative even at 10^10^ IVT RNA copies/µL. All IVTs were detected using the actin system at all tested concentrations.

Ct < 40 were plotted against the RNA copies/µL using GraphPad Prism and a linear regression was estimated (alongside 95% confidence intervals) for the four sequences detected by gTJ-seg2. The linear regression formulas were as follows: JMTV *y* = −3.878 x *x* + 44.72; YGTV *y* = −3.687 x *x* + 42.49; ALSV *y* = −3.410 x *x* + 42.99; PLJV *y* = −2.807 x *x* + 47.33, where *y* is Ct and *x* is log_10_(RNA copies/µL).

**Fig. 2:**
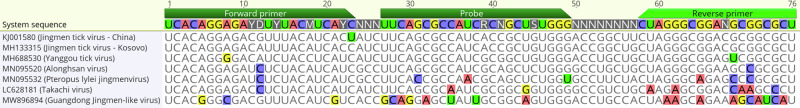
Multiple sequence alignment of the region amplified by gTJ-seg2 in seven reference jingmenvirus sequences of increasing genetic divergence. The colored nucleotides do not match the gTJ-seg2 sequences. Figure generated using Geneious Prime (Version 2023.4).

These results are in line with the in silico sequence analysis of these viruses over the region amplified by the segment 2 system (Fig. 2). Indeed, JMTV, YGTV and ALSV have either none or only one mismatch when comparing their sequence with the forward primer, the probe, or the reverse primer. In contrast, PLJV contains three mismatches with the probe and reverse primer, which could explain its higher limit of detection. TAKV presents six mismatches with the reverse primer which could explain why it cannot be amplified by gTJ-seg2. The divergent GJLV cannot be amplified either using gTJ-seg2, as it has 3 mismatches in the forward primer, 8 mismatches over the probe, and 10 mismatches over the reverse primer.

### Tick collection and morphological identification

In total, 6217 adult ticks and 52 nymphs were collected and 1715 pools were formed (Table 2). Overall, 3923 ticks from eight species were collected from 763 cattle, 365 were infested with ticks (45%), and morphologically identified. The most abundant species in cattle was *R. bursa* (*n* = 2310; 59.9%), followed by *Hyalomma marginatum* (*n* = 1036; 26.4%), *H. scupense* (*n* = 282; 7.1%), *R. annulatus* (*n* = 130; 3.3%), *Ixodes ricinus* (*n* = 75; 1.9%), *R. sanguineus* (*n* = 74; 1.9%), *Haemaphysalis punctata* (*n* = 14; 0.4%), and *Dermacentor marginatus* (*n* = 2). A total of 1682 ticks from three species were collected from 657 horses, the most abundant species was *H. marginatum* (*n* = 1026; 61%), followed by *R. bursa* (*n* = 629; 37.4%) and *R. sanguineus* (*n* = 27;1.6%). A total of 626 ticks from three species were collected from 218 wild boars, the main species was *D. marginatus* (*n* = 613; 97.9%), followed by *H. marginatum* (*n* = 12; 1.9%) and *R. bursa* (*n* = 1). A total of 38 *R. bursa* ticks were collected from sheep (Table 2).

**Table 2:** Ticks collected from farmed and wild animals by tick species and host species

	Cattle *n* (%)	Horse *n* (%)	Boar *n* (%)	Sheep *n* (%)	Total *n* (%)
*R. bursa*	2310 (59.9)	629 (37.4)	1 (0.2)	38 (100)	2978 (47.5)
*H. marginatum*	1036 (26.4)	1026 (61.0)	12 (1.9)	/	2074 (33.1)
*H. scupense*	282 (7.1)	/	/	/	282 (4.5)
*R. annulatus*	130 (3.3)	/	/	/	130 (2.1)
*I. ricinus*	75 (1.9)	/	/	/	75 (1.2)
*R. sanguineus*	74 (1.9)	27 (1.6)	/	/	101 (1.6)
*Hae. punctata*	14 (0.4)	/	/	/	14 (0.2)
*D. marginatus*	2 (0.05)	/	613 (97.9)	/	615 (9.8)
Total *n* (%)	3923 (62.6)	1682 (26.8)	626 (10.0)	38 (0.6)	6269

*R* Rhipicephalus, *H* Hyalomma, *I* Ixodes, *Hae* Haemaphysalis, *D* Dermacentor.

The overall adult male to female ratio in the collected ticks was 1.44. In cattle, that ratio was 1.72 (1421 females, 2452 males).

### Detection of tick-associated jingmenvirus RNA

In the 1,715 pools tested, tick-associated jingmenvirus RNA was detected in 21 tick pools collected from three cattle and in one tick pool collected from a sheep with a minimum infection rate of 3.51‰ (MIR, number of tick pools positive divided by the number of ticks tested reported as number of positive infections per 1000 tested ticks).

The highest prevalence was found in the *Rhipicephalus* genus with a MIR of 5.71‰ in *R. bursa* and 9.90‰ in *R. sanguineus* followed by *H. marginatum* 1.93‰. The MIR of positive ticks collected in cattle was 5.4‰, more specifically 6.93‰ for *R. bursa*, 1.35‰ for *R. sanguineus* and 3.86‰ for *H. marginatum*.

**Fig. 3:**
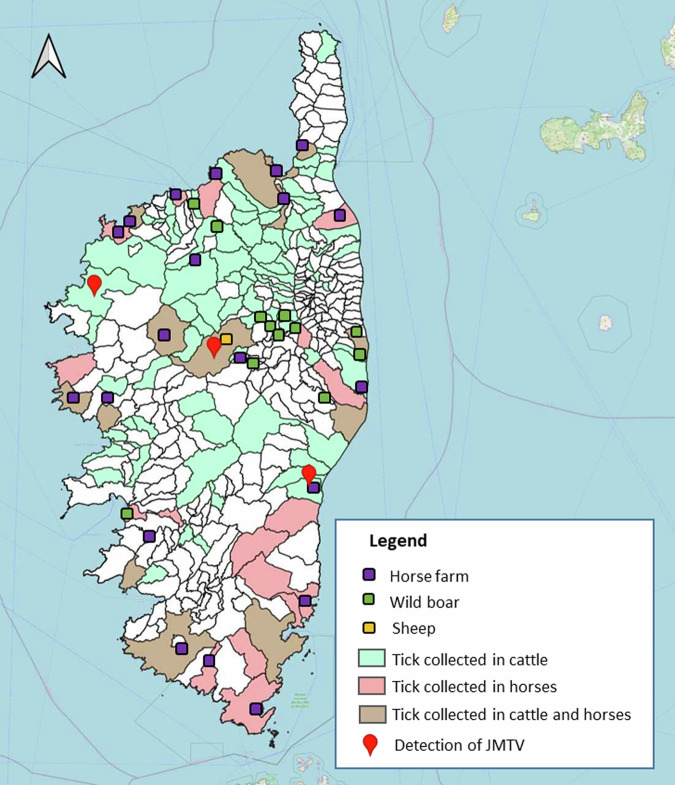
Map of Corsica indicating tick collection sites and positive detection of JMTV RNA in ticks of sites inspected. Color coded by vertebrate host.

Out of the 345 cattle infested with ticks, three (*n* = 345; 1%) had at least one positive tick pool. One bovine had 2/5 pools positive (40%), the second bovine had 13/27 pools positive (48%) and the last bovine had 6/14 pools positive (43%) (Table 3 and Supplementary Table 2). These three animals were from three distinct locations and breeding in Corsica (Fig. 3).

**Table 3:** gTJ-seg2-positive tick pool details

Host type	Host ID	Tick pool ID	Tick species	Ticks /pool	Sex	Location	Date	Ct	RNA copies/µL^a^
Cattle	3339	164BOV19^b^	*R bursa*	6	M	Ventiseri	22.05.19	16.32	3.4 × 10^7^
		165BOV19^c^	*R bursa*	6	M			30.68	1.0 × 10^4^
	2005	529BOV20	*R bursa*	6	M	Galeria	26.05.20	18.10	1.2 × 10^7^
		537BOV20^c^	*R bursa*	6	M			14.76	8.2 × 10^7^
		538BOV20^c^	*R bursa*	6	M			14.88	7.7 × 10^7^
		539BOV20^c^	*R bursa*	6	M			19.56	5.5 × 10^6^
		540BOV20^c^	*R bursa*	6	F			21.55	1.8 × 10^6^
		541BOV20^c^	*R bursa*	6	M			21.23	2.1 × 10^6^
		542BOV20^c^	*R bursa*	6	M			20.21	3.8 × 10^6^
		544BOV20^c^	*R bursa*	6	M			22.36	1.1 × 10^6^
		545BOV20^c^	*R sanguineus*	1	M			20.96	2.5 × 10^6^
		546BOV20^c^	*H marginatum*	6	M			22.66	9.6 × 10^5^
		547BOV20^c^	*H marginatum*	6	M			23.35	6.5 × 10^5^
		550BOV20^c^	*H marginatum*	5	M			21.45	1.9 × 10^6^
		552BOV20	*H marginatum*	3	F			31.78	5.6 × 10^3^
	2030	580BOV20^c^	*R bursa*	6	M	Not available^d^	26.05.20	34.51	1.2 × 10^3^
		581BOV20^c^	*R bursa*	6	M			30.15	1.4 × 10^4^
		582BOV20	*R bursa*	6	M			32.46	3.8 × 10^3^
		583BOV20	*R bursa*	6	M			33.29	2.4 × 10^3^
		584BOV20	*R bursa*	6	M			31.18	7.9 × 10^3^
		590BOV20	*R bursa*	4	M			30.96	8.9 × 10^3^
Sheep	20201	150SHE20^c^	*R bursa*	2	F	Corte	18.06.20	28.36	3.9 × 10^4^

^a^Estimated using the linear regression formula obtained for JMTV (see Fig. 1).

^b^Whole genome sequence obtained by random RNA NGS.

^c^Confirmed to be JMTV by NGS of RT-qPCR amplicon.

^d^The identification tag for this animal was lost before it could be examined for ticks.

Only two of the JMTV-positive pools collected from cattle were adult female ticks while the remaining (*n* = 19) pools were adult male ticks, indicating a significantly higher prevalence in male (0.79% [0.49%-1.12%]) than in female ticks (0.14% [0.02%-0.43%]) collected from cattle.

Only one of the 107 sampled sheep had a positive adult tick pool. That animal had 1/2 (50%) pools positive.

### Sequence and phylogenetic analyses

We obtained the full genome sequence of JMTV Corsica 164BOV19 from a pool of 6 male *R. bursa* ticks collected from cattle in 2020 (Table 3) (Genbank accession numbers PP275067-PP275070). The segment sequences shared ~95% nucleotide identity (94.0–95.3% depending on the strains and segments) with other European sequences of JMTV (clade II), detected in humans from Kosovo (MH133321-MH133324), and in *R. bursa* from Romania (MW561147-MW561150) and Türkiye (MN486256, MN486261, MN486264, MN486267). JMTV Corsica 164BOV19 was more distantly related (84.4-90.2% depending on the strains and segments) to three other clade II strains, detected in ticks from the French Antilles and Trinidad and Tobago or in a mosquito colony from Italy. JMTV Corsica 164BOV19 is even more distantly related (77.2–82.6% depending on the strains and segments) to sequences from clade I, of Asian, South American and African origins (Table 4). We obtained partial sequences for 15 positive samples, by NGS of RT-qPCR amplicons; all were 100% identical at the nucleotide level to the JMTV sequence obtained for 164BOV19.

**Table 4:** Percentage nucleotide identity over the open reading frames of the four genomic segments of selected published JMTV strains and JMTV Corsica (164BOV19, Genbank accession numbers PP275067-PP275070)

Location	Host	Strain	Clade	% nt identity (-segment)				Genbank accession numbers
				-1	-2	-3	-4	
Kosovo	Human	2015-A-K15-1A	II	94.8	95.1	94.8	94.6	MH133321-MH133324
Romania	Tick	Tulcea1	II	94.2	95.0	95.3	94.5	MW561147-MW561150
Türkiye	Tick	T14	II	94.0	94.7	94.7	94.5	MN486256, MN486261, MN486264, MN486267
French Antilles	Tick	JMTV/ *Rh. microplus*/ *Am. variegatum*/ French Antilles	II	86.2	87.4	87.6	87.5	MN095523-MN095526
Trinidad and Tobago	Tick	TTP-Pool-3b	II	85.0	90.2	86.9	86.9	MN025512-MN025515
Italy	Mosquito	RIMINI	II	84.4	87.1	87.1	86.8	BK059426-BK05942
Laos	Tick	JMTV/ *Am. testudinarium*/ Lao PDR	I	80.2	80.3	79.3	77.5	MN095527-MN095530
Japan	Tick	19EH-IM24	I	80.2	80.2	79.4	77.7	LC628156-LC628159
Brazil	Tick	MGTV/V4/11	I	80.0	79.6	79.2	77.9	JX390985, JX390986, KY523073, KY523074
China	Tick	SY84	I	79.4	81.1	79.2	77.2	KJ001579-KJ001582
Uganda	Monkey	RC27	I	79.3	82.6^a^	79.1^a^	77.9	KX377513-KX377516
Kenya	Tick	MT328	I	79.1	80.6	79.6	77.4	ON186499, ON186506, ON186513, ON186520
Guinea	Tick	KITV/2017/1	I	78.3	79.8	79.6	77.7	MK673133-MK673136

^a^Partial sequence.

**Fig. 4:**
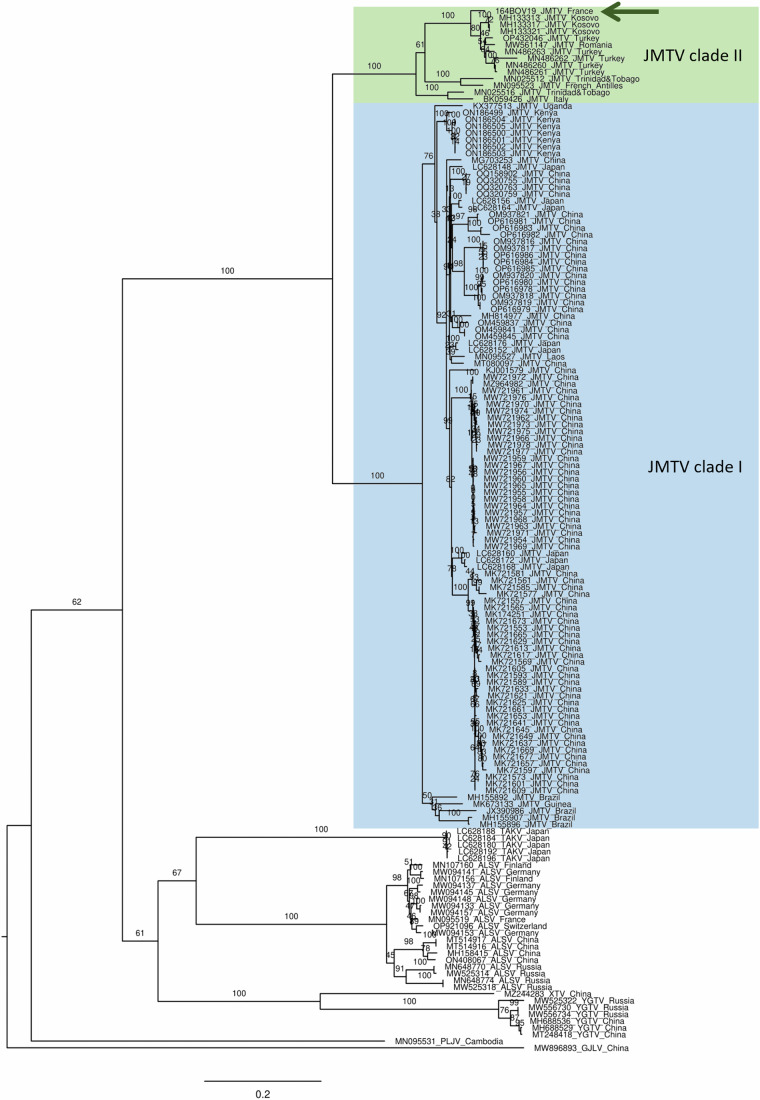
Maximum likelihood analysis of nucleotide sequences of segment 1 ORF of jingmenviruses from the tick-associated clade. The phylogenetic tree was constructed using the general time reversible model, gamma distributed with 100 bootstraps (branch labels). The scale bar represents the number of nucleotide substitution per site.

The JMTV Corsica 164BOV19 sequences were aligned with published jingmenvirus sequences and phylogenetic trees were built for each segment (Fig. 4 and Supplementary Figs. 1–3). We found that JMTV Corsica 164BOV19 was most closely related to European and Caribbean JMTV sequences (clade II), separately from Asian, South American, and African JMTV strains, which form another clade (clade I).

### Virus isolation

We attempted the isolation of JMTV in new-born mice by inoculating JMTV-positive tick homogenate intra-cranially in two to three days old OF1 mice. No clinical signs were observed in the inoculated mice for two weeks. While JMTV RNA was detectable in the inoculum, viral RNA was not detected in the mice brains fourteen days after intra-cranial inoculation using gTJ-seg2 in RT-qPCR, suggesting the lack of virus propagation.

## Discussion

In this study, we designed and characterized two RT-qPCR systems (gTJ-seg1 and gTJ-seg2) targeting segments 1 and 2 of the three tick-associated jingmenviruses published at the time (JMTV, ALSV, and YGTV) and we report evidence of circulation of JMTV in ticks collected from cattle in Corsica.

Segment 2 was chosen as a target for the detection and quantification of tick-associated jingmenviruses due to its relatively high homology between target species, the absence of homology with other viruses or living beings^1^ and the lower limit of detection of gTJ-seg2 compared to gTJ-seg1. We showed that gTJ-seg2 can detect several jingmenvirus sequences (JMTV, YGTV, ALSV and PLJV, but not TAKV or GLJV), all in the tick-associated jingmenvirus group. While some RT-qPCR assays have been developed to detect single species of jingmenviruses, to our knowledge, gTJ-seg2 is the first RT-qPCR assay to detect all aforementioned tick-associated jingmenviruses^27^.

Therefore, gTJ-seg2 assay was used to screen ticks, belonging to eight species, collected in Corsica, and successfully detected JMTV in 22 pools of *R. bursa*, *H. marginatum* and *R. sanguineus*, with the highest JMTV detection rate found in *R. bursa*. JMTV has previously been detected in these tick species from other regions: *R. bursa* from Türkiye and Romania, in *H. marginatum* from Türkiye and in *R. sanguineus* from Türkiye and China^1,10,11,28^. The varying tick numbers and different detection methods prevent direct comparisons with these studies. Taken together, published data to date and our findings seem to indicate an association between JMTV and *Rhipicephalus* ticks, while JMTV can be found in other tick genera, albeit in lower prevalence^10,11^. To date, this association has only been made through observation of higher prevalence in *R. microplus* cohorts but remains to be formally demonstrated with vector competence studies.

The RNA loads detected in Corsican ticks were higher than the limit of detection for JMTV, YGTV, and ALSV by a few orders of magnitude (up to 10^7^ RNA copies/µL detected vs a limit of detection around 10^2^ RNA copies/uL) suggesting that YGTV and ALSV would have been detected in those ticks, had they been present. PLJV might have been detected (limit of detection around 10^4^ RNA copies/µL) if present, although this sequence has only ever been detected in bat-derived samples. However, TAKV would not have been detected by our system and would require screening using a virus-specific detection system or a newly developed generic system, based on alignments including this more recently discovered sequence. Therefore, a limitation of this study is that we cannot conclude on the presence or absence of TAKV in our samples at this time.

We were not successful in isolating JMTV Corsica from new-born mice, despite inoculating each animal with >10^8^ viral RNA copies. JMTV isolation was previously reported as challenging in both new-born mice and cell culture^1,5^. Elucidating the mechanisms preventing JMTV propagation in laboratory models would allow for a better understanding of viral replication and host-restriction mechanisms and would be crucial in case they emerge as human or veterinary pathogens.

Many questions remain unanswered regarding the natural life cycle of tick-associated jingmenviruses, but there is mounting evidence that they are arthropod-borne viruses. Jingmenviruses have been detected both in male, female, engorged and non-engorged adult ticks as well as non-engorged larvae, which can be evidence for trans-ovarian transmission^18,29^. Our cattle-harvested JMTV-positive ticks corresponded to three animals, and these animals were in contact with up to 13 positive tick pools; this is not incompatible with the hypotheses that JTMV could be horizontally transmitted from the cattle to the ticks, or through co-feeding transmission from tick to tick, as previously described for tick-borne flaviviruses^3,30,31^. Another argument in favor of the horizontal transmission hypothesis is the presence of jingmenviruses in the salivary glands of ticks and in association with vertebrates, including humans presenting tick bites and febrile illness symptoms, monkeys, rodents, bats, pigs, sheep, cattle, and tortoises^5,17^. Moreover, there appears to be a lack of correlation between jingmenvirus sequences and their respective hosts of origin. Instead, phylogenetic clades and geographical distribution are notably associated^18,32^. This pattern is prominently evident in our phylogenetic analyses, incorporating JMTV Corsica within the Caribbean-European JMTV clade (clade II), distinct from the African-Asian-South American clade (clade I). Together, these data align with the hypothesis that jingmenviruses can be transmitted horizontally between ticks and vertebrate hosts, hypothesis which remains to be formally demonstrated with vector competence studies.

In light of the strong emerging potential of tick-associated jingmenviruses, the development of a molecular detection tool is important to improve the knowledge on virus circulation by implementing surveillance on vectors and reservoirs, as well as to encourage physicians to consider jingmenvirus infection in patients with undiagnosed febrile illness. Whether the gTJ-seg2 real-time molecular assay, developed in our study, is sensitive enough to be suited for diagnostics in clinical samples from human patients remains to be investigated. The need for serological techniques must also be evaluated. To the best of our knowledge, we provide here the first evidence of JMTV circulation in Corsica and more widely in the West Mediterranean region, and the first RT-qPCR system validated for the detection of multiple tick-associated jingmenviruses, which can be used as a screening tool for the surveillance of these viruses in arthropod or vertebrate hosts.

## Methods

### Molecular detection system design

We aligned all sequences of JMTV, ALSV, and YGTV available at the time of design (14 June 2021) from the taxonomy browser of the National Center of Biotechnology Information (NCBI) using the L-INS-I algorithm implemented in MAFFT version 7. Two RT-qPCR assays were designed targeting conserved regions of segments 1 and 2 based on the generated alignment (Table 5). In particular, segment 2 was selected due to its lack of homology with other virus sequences, in an effort to prevent non-specific amplification.

**Table 5:** Nucleotide sequences and amplicon size of two generic tick-associated jingmenvirus (gTJ) RT-qPCR systems designed on multiple sequence alignments of segments 1 (non-structural) and 2 (structural)

Name	Function	5′ to 3′ sequence	Amplicon size
gTJ-seg1 F	Forward	ATYACNGCYGTYTCYCTNTGGGA	92 bp
gTJ-seg1 R	Reverse	TTGACRTKYTTYAYRTTRGCRTTGAT	–
gTJ-seg1 P	Probe	FAM-TGGATGGCCGACCCYGCYATAA-Tamra	–
gTJ-seg2 F	Forward	TCACAGGAGAYDTYTACMTCAYC	76 bp
gTJ-seg2 R	Reverse	AGCGCCGCNTCCGCCCTAG	–
gTJ-seg2 P	Probe	FAM-TTCAGCGCCATCRCNGCTSTGGG-Tamra	–

### Molecular detection system selection

To ensure that the RT-qPCR detection systems targeting segments 1 and 2 were functional, a 0.1 µg/µL DNA plasmid synthetized by Geneart (ThermoFisher) containing the regions targeted by the systems in JMTV was serially diluted 10-fold from 100 pg/µL to 0.1 pg/µL and used as template in a qPCR assay (Superscript III, ThermoFisher).

### Generation of in vitro transcribed RNAs

In vitro transcripts were generated as positive controls and to evaluate the sensitivity and range of detection of the RT-qPCR systems. Plasmids were synthetized by Geneart containing the region of segment 2 amplified by the gTJ-seg2 system, for 6 jingmenviruses: JMTV (MH133315), ALSV (MN095520), YGTV (MH688530), TAKV (LC628181), PLJV (MN095532), and GJLV (MW896894) (sequences available in Supplementary Table 3). An exogenic NotI hybridization sequence was incorporated to detect potential laboratory contamination, and an actin sequence was also included as another target to quantify the IVT (see Supplementary Table 3). The plasmids were in vitro transcribed into RNA using Megashortscript^TM^ T7 transcription kit (Invitrogen ThermoFisher Scientific) with Turbo DNase to remove DNA. RNA transcripts were purified using MEGAclear^TM^ Purification of transcription reaction kit (Invitrogen ThermoFisher Scientific). A Thermo Scientific^TM^ NanoDrop^TM^ was used to determine the RNA concentration in ng/µL, converted to RNA copies/µL using the molecular weight of the molecule, calculated with the AAT Bioquest calculator online.

### Determining the range and limit of detection of the gTJ-seg2 system

The six IVTs were serially diluted 1:3 with dilutions ranging from 10^7^ RNA copies/µL to 15 RNA copies/µL, and used as templates in RT-qPCR using gTJ-seg2, with 4 to 8 replicates. All IVTs were tested with the actin system to confirm their reactivity. The cycle threshold (Ct) values obtained were graphed relative to the IVT concentration in logarithmic scale. A Ct value > 40 was considered negative. IVTs negative for all dilutions under 10^7^ RNA copies/µL were tested further, with dilutions starting from 10^10^ RNA copies/µL.

SPSS Statistics software version 24 (IBM) was used to determine the lower limit of detection (LOD), defined as the lowest concentration of RNA achieving a 95% hit rate (LOD95). GraphPad Prism 9.4.1 was used to estimate a linear regression and 95% confidence intervals between the Ct values < 40 obtained with gTJ-seg2 and the RNA copies/µL.

### RT-qPCR

RT-qPCR was performed using QuantiFast Probe RT-PCR kit (Qiagen), on a QuantStudio^TM^ 3 real-time PCR system, software version “V1.5.1”. Primers and probes were synthetized by Sigma-Aldrich, Merck KGaA. Reactions were set up with 12.5 µL buffer, 0.25 µL RT enzyme, 0.5 µL Rox, 800 nM of each primer, and 200 nM of probe for a final volume of 25 µL. The optimal cycling conditions were: 50 °C for 10 min; 95 °C for 5 min; 45 cycles of 95 °C for 10 s, and Tm for 30 s, with Tm = 57 °C for segment 1, Tm = 55 °C for segment 2, and Tm = 60 °C for actin. Probes were labeled with FAM dye and Tamra quencher.

### Sample collection

Several types of vertebrate animals were inspected for adult ticks between August 2018 and June 2020, all over Corsica (Fig. 3); 763 bovine cattle from various farms were inspected from January 2019 to June 2020 at the main active slaughterhouse in Corsica, in Ponte-Leccia and; 657 horses were inspected several times between March and August 2019, and in May and June 2020, after each riding excursion in the natural environment across Corsica. Ticks were collected from 218 wild boars in the northeast of Corsica from August to December (hunting season) in 2018 and 2019. A total of 107 sheep were inspected monthly for ticks in May and in June 2020 in a farm located in Corte (Fig. 3).

All ticks were collected and kept alive until morphological identification^33^ and storage. Ticks were identified at species level under a stereomicroscope using an identification key^33^, and immediately stored at –80°C.

### Sample processing and nucleic acid extraction

Ticks collected from the animals were washed once in 70% ethanol and twice in distilled water, then were divided either individually or in monospecific pools of up to 10 ticks, according to developmental stage, sex, and animal of collection. They were homogenized in Minimum Essential Medium (MEM) containing 15% of fetal bovine serum, antibiotics (1% penicillin-streptomycin, 1% kanamycin), fungicide (5% amphotericin B), and 1% L-glutamine, using a TissueLyser II (Qiagen, Hilden, Germany) at 30 Hz for 3 min, for nucleic acid extraction and to allow viral isolation attempts. Nucleic acid extractions were performed on a QIAcube HT (Qiagen) using QIAamp cador Pathogen Mini kits (Qiagen), according to the manufacturer’s instructions. Nucleic acid extracts were eluted in 100 μL buffer and stored at -80°C until they were used as templates in RT-qPCR. The extraction quality was monitored by systematically spiking MS2 bacteriophage and quantifying it by RT-qPCR.

### Complete genome and RT-qPCR amplicon sequencing

One gTJ-seg2-positive pool containing 6 male *R. bursa* ticks (164BOV19) was selected to obtain a complete genome using next-generation sequencing (NGS). A total of 200 μL homogenate supernatant was incubated at 37°C for 7 h with 25 U of Benzonase (Novagen) and MgCl_2_. RNA extraction was performed using the Viral RNA mini kit (Qiagen) on the BioRobot EZ1-XL Advanced (Qiagen). Random two-step RT-PCR was performed using tagged random primers in a ProtoScript® II Reverse Transcriptase (New England Biolabs) reaction followed by a Platinum® Taq High Fidelity polymerase (ThermoFisher Scientific) reaction with specific primers^34^. These samples and selected RT-qPCR amplicons produced as described above using gTJ-seg2 were quantified using the Qubit® dsDNA HS Assay Kit and a Qubit 2.0 fluorometer (ThermoFisher Scientific). All amplicons were sonicated into 200 bp fragments and libraries were built and barcoded using AB Library Builder System (ThermoFisher Scientific). The barcoded fragments were quantified by RT-qPCR using the Ion Library TaqMan™ Quantitation Kit (ThermoFisher Scientific) and pooled equimolarly. An emulsion PCR of the pooled fragments was performed and the samples were loaded on an Ion 520 chip (ThermoFisher Scientific) using an automated Ion Chef instrument (ThermoFisher Scientific). Sequencing was performed using the S5 Ion torrent technology (ThermoFisher Scientific) following the manufacturer’s instructions. Reads were trimmed (reads with quality score <0.99 or length <100pb were removed, and the first and last 30 nucleotides were removed from all reads) and de novo contigs were generated with CLC Genomics Workbench software v.21 (Qiagen). These contigs were blasted (in house BLASTn algorithm) to determine the best reference sequence, and reads were then mapped to that sequence. Parameters for reference-based assembly consisted of match score = 1, mismatch cost = 2, length fraction = 0.5, similarity fraction = 0.8, insertion cost = 3, and deletion cost = 3.

### Phylogenetic analyses

The complete nucleotide sequences of JMTV Corsica 164BOV19 four genome segments obtained in this study were aligned with published tick-associated jingmenvirus sequences using the E-INS-I algorithm implemented in MAFFT version 7^35^. Phylogenetic analyses were inferred for each genomic segment, with maximum likelihood in Mega X^36^, with 100 bootstraps, a general time reversible model gamma distributed.

### Isolation attempts of virus

A total of 10 µL of homogenized tick pool 164BOV19, diluted 1:10 with MEM was injected intracerebrally into 2-day-old OF1 mice (*n* = 15). This corresponded to a dose of 2.3 × 10^8 RNA copies per animal. The mice were observed for 14 days for clinical signs of disease, then euthanized by cervical dislocation under general anesthesia, and brains were collected. Nucleic acids were purified from the brain tissues using QIAcube HT with QIAamp 96 Virus QIAcube HT Kit (Qiagen), and tested for presence of JMTV with the segment 2 RT-qPCR described above.

In vivo experiments were approved by the French ‘Ministère de l’Enseignement Supérieur, de la Recherche et de l’Innovation’ (Autorisation de projet utilisant des animaux à des fins scientifiques #9368) and performed in accordance with the French national guidelines and the European legislation covering the use of animals for scientific purposes. All experiments were conducted in a BSL3 laboratory.

## Supplementary information

Supplementary Information

## Data Availability

The sequences of JMTV Corsica 164BOV19 genomic segments were deposited on Genbank under accession numbers PP275067-PP275070.
