# Supplementary material for: First detection of Jingmen tick virus in Corsica with a new generic RTqPCR system
Source: Npj Viruses. 2024 Sep 30;2:44. doi: 10.1038/s44298-024-00053-1 (PMC11721387; doi:10.1038/s44298-024-00053-1)
Supplement: Supplementary file 1 — Supplementary Information [file 44298_2024_53_MOESM1_ESM.pdf]

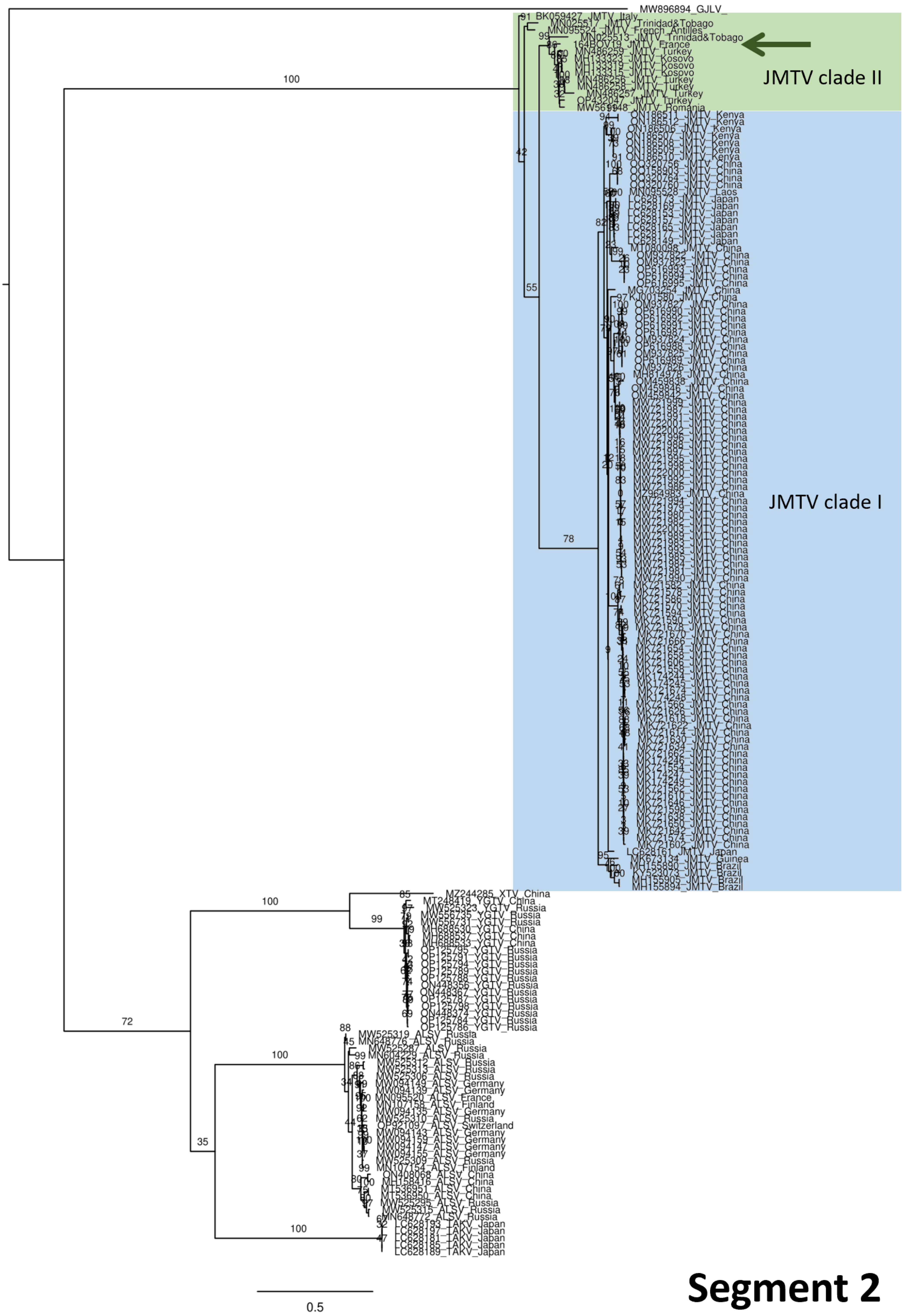

Supplementary Figure 1: Maximum likelihood analysis of nucleotide sequences of segment 2 ORF of jingmenviruses from the tick-associated clade. The phylogenetic tree was constructed using the general time reversible model, gamma distributed with 100 bootstraps (branch labels). The scale bar represents the number of nucleotide substitution per site.

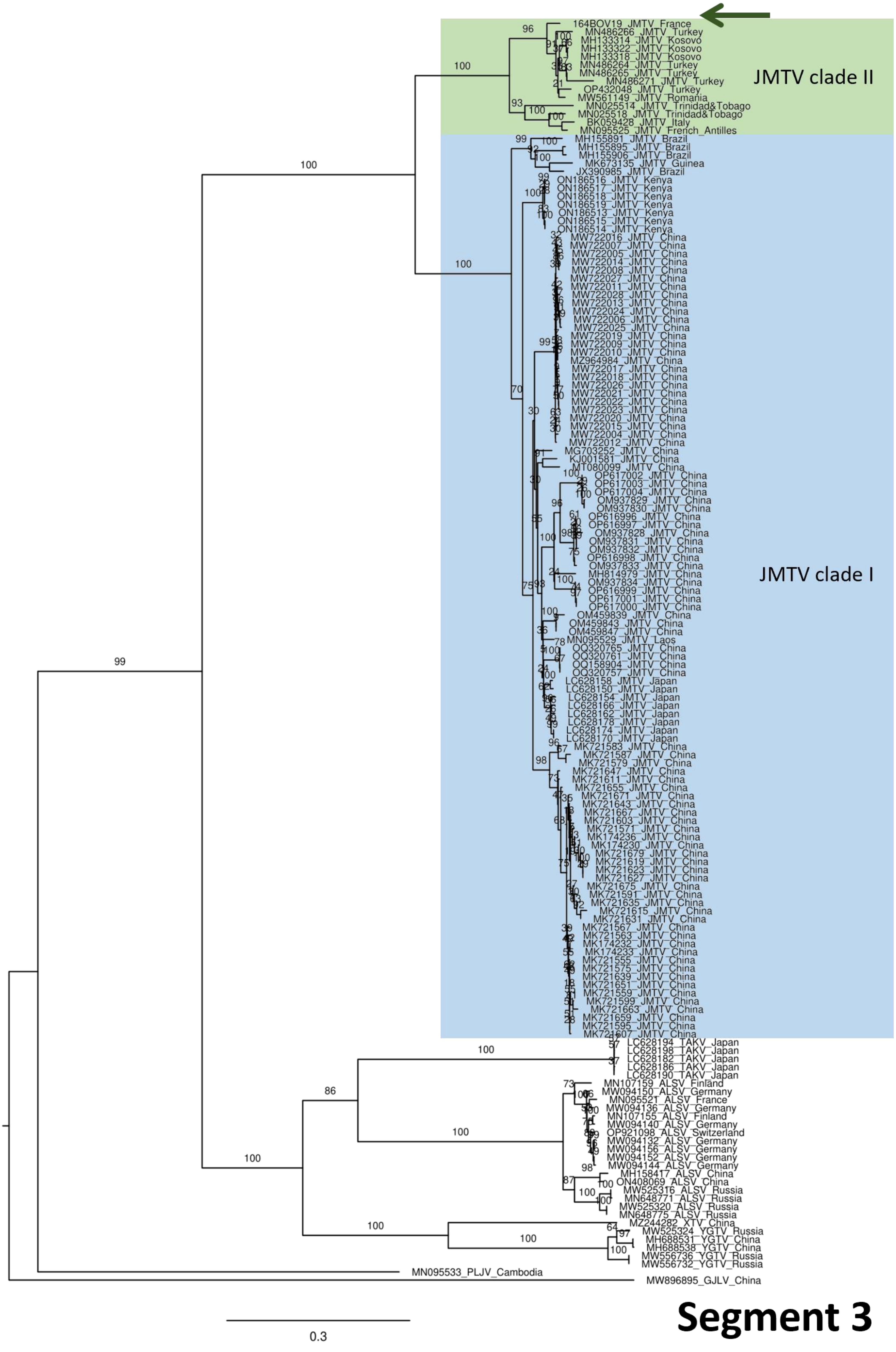

Supplementary Figure 2: Maximum likelihood analysis of nucleotide sequences of segment 3 ORF of jingmenviruses from the tick-associated clade. The phylogenetic tree was constructed using the general time reversible model, gamma distributed with 100 bootstraps (branch labels). The scale bar represents the number of nucleotide substitution per site.

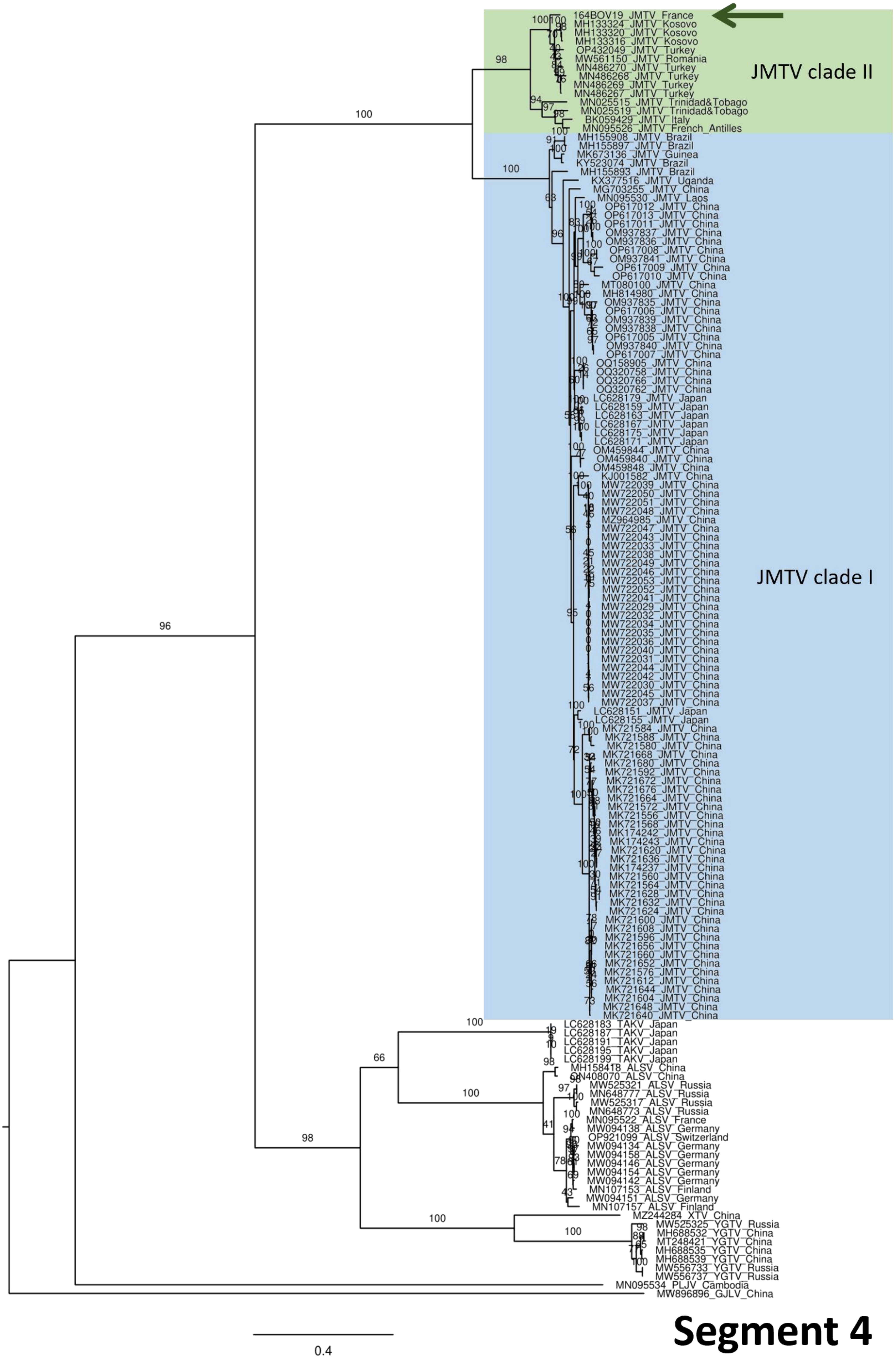

Supplementary Figure 3: Maximum likelihood analysis of nucleotide sequences of segment 4 ORF of jingmenviruses from the tick-associated clade. The phylogenetic tree was constructed using the general time reversible model, gamma distributed with 100 bootstraps (branch labels). The scale bar represents the number of nucleotide substitution per site.

Supplementary Table 1: RT-qPCR results using various dilutions of IVT from six increasingly divergent jingmenvirus sequences as template, with gTJ-seg2 and actin systems.

gTJ-seg2	JMTV		YGTV		ALSV		PLJV		TAKV		GJLV	
IVT RNA copies/µL	Positives/replicates (%)	Mean positive CT (SD)	Positives/replicates (%)	Mean positive CT (SD)	Positives/replicates (%)	Mean positive CT (SD)	Positives/replicates (%)	Mean positive CT (SD)	Positives/replicates (%)	Mean positive CT (SD)	Positives/replicates (%)	Mean positive CT (SD)
10000000000	ND	ND	ND	ND	ND	ND	ND	ND	0/4 (0)	N/A	0/4 (0)	N/A
3333333333	ND	ND	ND	ND	ND	ND	ND	ND	0/4 (0)	N/A	0/4 (0)	N/A
1111111111	ND	ND	ND	ND	ND	ND	ND	ND	0/4 (0)	N/A	0/4 (0)	N/A
370370370	ND	ND	ND	ND	ND	ND	ND	ND	0/4 (0)	N/A	0/4 (0)	N/A
123456790	ND	ND	ND	ND	ND	ND	ND	ND	0/4 (0)	N/A	0/4 (0)	N/A
41152263	ND	ND	ND	ND	ND	ND	ND	ND	0/4 (0)	N/A	0/4 (0)	N/A
13717421	ND	ND	ND	ND	ND	ND	ND	ND	0/4 (0)	N/A	0/4 (0)	N/A
2631579	4/4 (100)	20.8 (0.4)	4/4 (100)	19.9 (0.6)	4/4 (100)	22.0 (0.8)	4/4 (100)	29.7 (1.4)	ND	ND	ND	ND
877193	4/4 (100)	21.9 (0.5)	4/4 (100)	20.8 (0.5)	4/4 (100)	22.9 (0.5)	4/4 (100)	30.6 (1.3)	0/4 (0)	N/A	0/4 (0)	N/A
292398	4/4 (100)	23.2 (0.4)	4/4 (100)	21.8 (0.1)	4/4 (100)	23.7 (0.4)	4/4 (100)	31.6 (1.4)	0/4 (0)	N/A	0/4 (0)	N/A
97466	4/4 (100)	24.9 (0.3)	4/4 (100)	23.4 (0.5)	4/4 (100)	25.3 (0.5)	4/4 (100)	33.1 (1.0)	0/4 (0)	N/A	0/4 (0)	N/A
32489	4/4 (100)	26.8 (0.5)	4/4 (100)	25.9 (0.5)	4/4 (100)	27.3 (0.6)	4/4 (100)	34.8 (1.4)	0/4 (0)	N/A	0/4 (0)	N/A
10830	4/4 (100)	28.8 (0.6)	4/4 (100)	27.1 (1.0)	4/4 (100)	29.3 (0.7)	3/4 (75)	36.3 (1.2)	ND	ND	ND	ND
3610	4/4 (100)	30.8 (0.5)	4/4 (100)	29.0 (1.2)	4/4 (100)	31.0 (0.5)	0/4 (0)	N/A	ND	ND	ND	ND
1203	4/4 (100)	32.0 (1.1)	4/4 (100)	31.2 (1.1)	4/4 (100)	32.6 (0.2)	0/4 (0)	N/A	ND	ND	ND	ND
401	4/4 (100)	35.1 (0.7)	4/4 (100)	32.7 (1.6)	4/4 (100)	34.2 (1.8)	0/4 (0)	N/A	ND	ND	ND	ND
134	3/4 (75)	37.4 (1.0)	4/4 (100)	35.5 (1.8)	3/4 (75)	36.0 (1.4)	0/4 (0)	N/A	ND	ND	ND	ND
44.6	1/4 (25)	39.0 (N/A)	2/4 (50)	37.0 (2.1)	0/4 (0)	N/A	0/4 (0)	N/A	ND	ND	ND	ND
14.9	0/4 (0)	N/A	0/4 (0)	N/A	0/4 (0)	N/A	0/4 (0)	N/A	ND	ND	ND	ND

Actin	JMTV		YGTV		ALSV		PLJV		TAKV		GJLV	
IVT RNA copies/µL	Positives/replicates (%)	Mean positive CT (SD)	Positives/replicates (%)	Mean positive CT (SD)	Positives/replicates (%)	Mean positive CT (SD)	Positives/replicates (%)	Mean positive CT (SD)	Positives/replicates (%)	Mean positive CT (SD)	Positives/replicates (%)	Mean positive CT (SD)
10000000	8/8 (100)	16.1 (0.4)	8/8 (100)	18.1 (0.5)	8/8 (100)	18.8 (0.4)	8/8 (100)	17.9 (0.2)	8/8 (100)	16.9 (0.3)	8/8 (100)	16.2 (0.5)
3333333	8/8 (100)	17.8 (0.2)	8/8 (100)	19.6 (0.2)	8/8 (100)	19.6 (0.5)	8/8 (100)	19.3 (0.2)	8/8 (100)	18.0 (0.2)	8/8 (100)	18.2 (0.4)
1111111	8/8 (100)	19.3 (0.4)	8/8 (100)	21.4 (0.2)	8/8 (100)	20.9 (0.3)	8/8 (100)	20.9 (0.1)	8/8 (100)	19.3 (0.2)	8/8 (100)	20.0 (0.4)
370370	8/8 (100)	21.5 (0.2)	8/8 (100)	23.2 (0.3)	8/8 (100)	22.7 (0.1)	8/8 (100)	22.7 (0.2)	8/8 (100)	21.1 (0.2)	8/8 (100)	21.8 (0.5)
123457	8/8 (100)	23.2 (0.2)	8/8 (100)	24.8 (0.2)	8/8 (100)	24.8 (0.2)	8/8 (100)	24.5 (0.2)	8/8 (100)	23.0 (0.4)	8/8 (100)	23.4 (0.5)
41152	8/8 (100)	25.0 (0.3)	8/8 (100)	26.4 (0.6)	8/8 (100)	26.3 (0.2)	8/8 (100)	26.0 (0.3)	8/8 (100)	24.7 (0.4)	8/8 (100)	25.5 (1.0)
13717	8/8 (100)	26.8 (0.3)	8/8 (100)	28.0 (0.3)	8/8 (100)	27.7 (0.4)	8/8 (100)	27.7 (0.2)	8/8 (100)	26.1 (0.3)	8/8 (100)	26.5 (0.3)
4572	8/8 (100)	28.2 (0.5)	8/8 (100)	29.3 (0.3)	8/8 (100)	29.3 (0.4)	8/8 (100)	29.0 (0.4)	8/8 (100)	27.6 (0.4)	8/8 (100)	28.1 (0.4)
1524	8/8 (100)	29.8 (0.9)	8/8 (100)	30.5 (1.0)	8/8 (100)	30.1 (0.4)	8/8 (100)	30.1 (0.6)	8/8 (100)	29.0 (0.6)	8/8 (100)	29.0 (0.4)
508	8/8 (100)	30.9 (0.8)	7/8 (87.5)	31.7 (0.5)	8/8 (100)	31.5 (0.6)	8/8 (100)	31.4 (0.9)	8/8 (100)	30.4 (0.7)	8/8 (100)	30.1 (0.7)
169	8/8 (100)	32.2 (1.7)	8/8 (100)	33.6 (0.7)	7/8 (87.5)	33.0 (0.8)	8/8 (100)	33.0 (0.7)	8/8 (100)	32.0 (0.6)	8/8 (100)	32.1 (0.7)
56	8/8 (100)	35.3 (0.7)	6/8 (75)	35.1 (0.4)	6/8 (75)	34.9 (1.4)	5/8 (62.5)	36.0 (1.8)	7/8 (87.5)	33.6 (1.4)	8/8 (100)	33.3 (1.0)

Supplementary Table 2: Distribution of tick species by host and gTJ-seg2 positive pools.

Number of Pools with *n* Ticks										Number of positive pools detected with gTJ-seg2				
**Cattle 2019 (n=456)**														
Number of Individual Ticks or Ticks per Pool (*n*)	*R. bursa*	*H. marginatum*	*H. scupense*	*H. punctata*	*R. sanguineus*	*B. annulatus*	*I. ricinus*	*D. marginatus*	**Total**	**Pool**	*R. bursa*	*H. marginatum*	*R sanguineus*	**Total**
**1**	60	55	30	4	3	12	6	0	**170**	**1**	0	0	0	0
**2**	25	25	6	0	0	2	5	0	**63**	**2**	0	0	0	0
**3**	14	13	6	0	1	4	3	0	**41**	**3**	0	0	0	0
**4**	17	10	4	0	0	0	1	0	**32**	**4**	0	0	0	0
**5**	12	5	2	0	0	4	0	0	**23**	**5**	0	0	0	0
**6**	30	16	11	0	0	5	5	0	**67**	**6**	2	0	0	2
**Total**	**158**	124	59	4	4	27	20	0	**396**	**Total**	2	0	0	**2**
**Number of ticks**	460	305	152	4	6	78	59	0	**1064**	**MLE (95% CI)**	**0.44 (0.07-1.35)**			**0.19 (0.03-0.58)**

Number of Pools with *n* Ticks										Number of positive pools detected with gTJ-seg2				
**Cattle 2020 (n=307)**														
Number of Individual Ticks or Ticks per Pool (*n*)	*R. bursa*	*H. marginatum*	*H. scupense*	*H. punctata*	*R. sanguineus*	*B. annulatus*	*I. ricinus*	*D. marginatus*	**Total**	**Pool**	*R. bursa*	*H. marginatum*	*R sanguineus*	**Total**
**1**	59	60	10	3	11	7	6	2	**158**	**1**	0	0	1	1
**2**	32	39	7	2	4	4	0	0	**88**	**2**	0	0	0	0
**3**	29	31	1	1	6	4	2	0	**74**	**3**	0	1	0	1
**4**	27	33	4	0	4	0	1	0	**69**	**4**	1	0	0	1
**5**	22	16	3	0	3	5	0	0	**49**	**5**	0	1	0	1
**6**	237	48	12	0	0	0	0	0	**297**	**6**	13	2	0	15
**Total**	406	227	37	6	28	20	9	2	**735**	**Total**	14	4	1	**19**
**Number of ticks**	1 850	731	130	10	68	52	16	2	**2 859**	**MLE (95% CI)**	**0.77 (0.44-1.25)**	**0.55 (0.17-1.28)**	**1.25 (0.07-5.38)**	**0.67 (0.42-1.02)**

Number of Pools with *n* Ticks										Number of positive pools detected with gTJ-seg2				
**Cattle 2019-2020 (n=763)**														
Number of Individual Ticks or Ticks per Pool (*n*)	*R. bursa*	*H. marginatum*	*H. scupense*	*H. punctata*	*R. sanguineus*	*B. annulatus*	*I. ricinus*	*D. marginatus*	**Total**	**Pool**	*R. bursa*	*H. marginatum*	*R sanguineus*	**Total**
**1**	119	115	40	7	14	19	12	2	**328**	**1**	0	0	1	1
**2**	57	64	13	2	4	6	5	0	**151**	**2**	0	0	0	0
**3**	43	44	7	1	7	8	5	0	**115**	**3**	0	1	0	1
**4**	44	43	8	0	4	0	2	0	**101**	**4**	1	0	0	1
**5**	34	21	5	0	3	9	0	0	**72**	**5**	0	1	0	1
**6**	267	64	23	0	0	5	5	0	**364**	**6**	15	2	0	17
**Total**	564	351	96	10	32	47	29	2	**1131**	**Total**	16	4	1	**21**
**Number of ticks**	2 310	1 036	282	14	74	130	75	2	**3 923**	**MLE (95% CI)**	**0.70 (0.41-1.11)**	**0.39 (0.12-0.9)**	**1,16 (0.07-5,01)**	**0.54 (0.34-0.81)**

Number of Pools with *n* Ticks										Number of positive pools detected with gTJ-seg2				
**Horse 2019 (n=538)**														
Number of Individual Ticks or Ticks per Pool (*n*)	*R. bursa*	*H. marginatum*	*H. scupense*	*H. punctata*	*R. sanguineus*	*B. annulatus*	*I. ricinus*	*D. marginatus*	**Total**	**Pool**	*R. bursa*	*H. marginatum*	*R sanguineus*	**Total**
**1**	15	29	0	0	0	0	0	0	**44**	**1**	0	0	0	0
**2**	10	12	0	0	0	0	0	0	**22**	**2**	0	0	0	0
**3**	9	12	0	0	0	0	0	0	**21**	**3**	0	0	0	0
**4**	9	14	0	0	0	0	0	0	**23**	**4**	0	0	0	0
**5**	4	12	0	0	0	0	0	0	**16**	**5**	0	0	0	0
**6**	5	16	0	0	0	0	0	0	**21**	**6**	0	0	0	0
**7**	5	11	0	0	0	0	0	0	**16**	**7**	0	0	0	0
**8**	6	12	0	0	0	0	0	0	**18**	**8**	0	0	0	0
**9**	3	7	0	0	0	0	0	0	**10**	**9**	0	0	0	0
**10**	32	17	0	0	0	0	0	0	**49**	**10**	0	0	0	0
**Total**	98	142	0	0	0	0	0	0	**240**	**Total**	0	0	0	0
**Number of ticks**	578	707	0	0	0	0	0	0	**1285**					

Number of Pools with *n* Ticks										Number of positive pools detected with gTJ-seg2				
**Horse 2020 (n=119)**														
Number of Individual Ticks or Ticks per Pool (*n*)	*R. bursa*	*H. marginatum*	*H. scupense*	*H. punctata*	*R. sanguineus*	*B. annulatus*	*I. ricinus*	*D. marginatus*	**Total**	**Pool**	*R. bursa*	*H. marginatum*	*R sanguineus*	**Total**
**1**	27	37	0	0	1	0	0	0	**65**	**1**	0	0	0	0
**2**	4	14	0	0	3	0	0	0	**21**	**2**	0	0	0	0
**3**	2	11	0	0	4	0	0	0	**17**	**3**	0	0	0	0
**4**	0	14	0	0	2	0	0	0	**16**	**4**	0	0	0	0
**5**	2	9	0	0	0	0	0	0	**11**	**5**	0	0	0	0
**6**	0	20	0	0	0	0	0	0	**20**	**6**	0	0	0	0
**7**	0	0	0	0	0	0	0	0	**0**	**7**	0	0	0	0
**8**	0	0	0	0	0	0	0	0	**0**	**8**	0	0	0	0
**9**	0	0	0	0	0	0	0	0	**0**	**9**	0	0	0	0
**10**	0	0	0	0	0	0	0	0	**0**	**10**	0	0	0	0
**Total**	35	105	0	0	10	0	0	0	**150**	**Total**	0	0	0	0
**Number of ticks**	51	319	0	0	27	0	0	0	**397**					

Number of Pools with *n* Ticks										Number of positive pools detected with gTJ-seg2				
**Horse 2019-2020 (n=657)**														
Number of Individual Ticks or Ticks per Pool (*n*)	*R. bursa*	*H. marginatum*	*H. scupense*	*H. punctata*	*R. sanguineus*	*B. annulatus*	*I. ricinus*	*D. marginatus*	**Total**	**Pool**	*R. bursa*	*H. marginatum*	*R sanguineus*	**Total**
**1**	42	66	0	0	1	0	0	0	**109**	**1**	0	0	0	0
**2**	14	26	0	0	3	0	0	0	**43**	**2**	0	0	0	0
**3**	11	23	0	0	4	0	0	0	**38**	**3**	0	0	0	0
**4**	9	28	0	0	2	0	0	0	**39**	**4**	0	0	0	0
**5**	6	21	0	0	0	0	0	0	**27**	**5**	0	0	0	0
**6**	5	36	0	0	0	0	0	0	**41**	**6**	0	0	0	0
**7**	5	11	0	0	0	0	0	0	**16**	**7**	0	0	0	0
**8**	6	12	0	0	0	0	0	0	**18**	**8**	0	0	0	0
**9**	3	7	0	0	0	0	0	0	**10**	**9**	0	0	0	0
**10**	32	17	0	0	0	0	0	0	**49**	**10**	0	0	0	0
**Total**	133	247	0	0	10	0	0	0	**390**	**Total**	0	0	0	0
**Number of ticks**	629	1026	0	0	27	0	0	0	**1682**					

Wild boars 2019										Number of positive pools detected with gTJ-seg2				
**Wild boars 2019 (n=157)**														
Number of Individual Ticks or Ticks per Pool (*n*)	*R. bursa*	*H. marginatum*	*H. scupense*	*H. punctata*	*R. sanguineus*	*B. annulatus*	*I. ricinus*	*D. marginatus*	**Total**	**Pool**	*R. bursa*	*H. marginatum*	*R sanguineus*	**Total**
1	1	0	0	0	0	0	0	18	19	1	0	0	0	0
**2**	0	0	0	0	0	0	0	15	**15**	**2**	0	0	0	0
**3**	0	0	0	0	0	0	0	11	**11**	**3**	0	0	0	0
**4**	0	0	0	0	0	0	0	14	**14**	**4**	0	0	0	0
**5**	0	0	0	0	0	0	0	10	**10**	**5**	0	0	0	0
**6**	0	0	0	0	0	0	0	42	**42**	**6**	0	0	0	0
**Total**	1	0	0	0	0	0	0	110	**111**	**Total**	0	0	0	0
**Number of ticks**	1	0	0	0	0	0	0	439	**440**					

Number of Pools with *n* Ticks										Number of positive pools detected with gTJ-seg2				
**Wild boars 2020 (n=61)**														
Number of Individual Ticks or Ticks per Pool (*n*)	*R. bursa*	*H. marginatum*	*H. scupense*	*H. punctata*	*R. sanguineus*	*B. annulatus*	*I. ricinus*	*D. marginatus*	**Total**	**Pool**	*R. bursa*	*H. marginatum*	*R sanguineus*	**Total**
**1**	0	2	0	0	0	0	0	10	**12**	1	0	0	0	0
**2**	0	2	0	0	0	0	0	2	**4**	**2**	0	0	0	0
**3**	0	2	0	0	0	0	0	9	**11**	**3**	0	0	0	0
**4**	0	0	0	0	0	0	0	4	**4**	**4**	0	0	0	0
**5**	0	0	0	0	0	0	0	3	**3**	**5**	0	0	0	0
**6**	0	0	0	0	0	0	0	17	**17**	**6**	0	0	0	0
**Total**	0	6	0	0	0	0	0	45	**51**	**Total**	0	0	0	0
**Number of ticks**	0	12	0	0	0	0	0	174	**186**					

Number of Pools with *n* Ticks										Number of positive pools detected with gTJ-seg2				
**Wild boars 19-2020 (n=218)**														
Number of Individual Ticks or Ticks per Pool (*n*)	*R. bursa*	*H. marginatum*	*H. scupense*	*H. punctata*	*R. sanguineus*	*B. annulatus*	*I. ricinus*	*D. marginatus*	**Total**	**Pool**	*R. bursa*	*H. marginatum*	*R sanguineus*	**Total**
**1**	1	2	0	0	0	0	0	28	**31**	**1**	0	0	0	0
**2**	0	2	0	0	0	0	0	17	**19**	**2**	0	0	0	0
**3**	0	2	0	0	0	0	0	20	**22**	**3**	0	0	0	0
**4**	0	0	0	0	0	0	0	18	**18**	**4**	0	0	0	0
**5**	0	0	0	0	0	0	0	13	**13**	**5**	0	0	0	0
**6**	0	0	0	0	0	0	0	59	**59**	**6**	0	0	0	0
**Total**	1	6	0	0	0	0	0	155	**162**	**Total**	0	0	0	0
**Number of ticks**	1	12	0	0	0	0	0	613	**626**					

Number of Pools with *n* Ticks										Number of positive pools detected with gTJ-seg2				
**Sheep 2019-2020 (n= 107)**														
Number of Individual Ticks or Ticks per Pool (*n*)	*R. bursa*	*H. marginatum*	*H. scupense*	*H. punctata*	*R. sanguineus*	*B. annulatus*	*I. ricinus*	*D. marginatus*	**Total**	**Pool**	*R. bursa*	*H. marginatum*	R sanguineus	**Total**
**1**	30	0	0	0	0	0	0	0	**30**	**1**	0	0	0	0
**2**	1	0	0	0	0	0	0	0	**1**	**2**	1	0	0	1
**3**	0	0	0	0	0	0	0	0	**0**	**3**	0	0	0	0
**4**	0	0	0	0	0	0	0	0	**0**	**4**	0	0	0	0
**5**	0	0	0	0	0	0	0	0	**0**	**5**	0	0	0	0
**6**	1	0	0	0	0	0	0	0	**1**	**6**	0	0	0	0
**Total**	32	0	0	0	0	0	0	0	**32**	**Total**	1	0	0	**1**
**Number of ticks**	38	0	0	0	0	0	0	0	38	**MLE (95% CI)**	**1.63 (0.09-6.96)**			**1.63 (0.09-6.96)**

Number of Pools with *n* Ticks										Number of positive pools detected with gTJ-seg2				
**Total 2019-2020**														
Number of Individual Ticks or Ticks per Pool (*n*)	*R. bursa*	*H. marginatum*	*H. scupense*	*H. punctata*	*R. sanguineus*	*B. annulatus*	*I. ricinus*	*D. marginatus*	**Total**	**Pool**	*R. bursa*	*H. marginatum*	*R sanguineus*	**Total**
**1**	192	183	40	7	15	19	12	30	498	**1**	0	0	1	1
**2**	72	92	13	2	7	6	5	17	214	**2**	1	0	0	1
**3**	54	69	7	1	11	8	5	20	175	**3**	0	1	0	1
**4**	53	71	8	0	6	0	2	18	158	**4**	1	0	0	1
**5**	40	42	5	0	3	9	0	13	112	**5**	0	1	0	1
**6**	273	100	23	0	0	5	5	59	465	**6**	15	2	0	17
**7**	5	11	0	0	0	0	0	0	16	**7**	0	0	0	0
**8**	6	12	0	0	0	0	0	0	18	**8**	0	0	0	0
**9**	3	7	0	0	0	0	0	0	10	**9**	0	0	0	0
**10**	32	17	0	0	0	0	0	0	49	**10**	0	0	0	0
**Total**	730	604	96	10	42	47	29	157	1715	**Total**	17	4	1	**22**
**Number of ticks**	2 978	2 074	282	14	101	130	75	615	6 269	**MLE (95% CI)**	**0.58 (0.35-0.9)**	**0.19 (0.06-0.45)**	**0.55 (0.03-2.41)**	**0.35 (0.23-0.52)**

Supplementary Table 3: *In vitro* transcript sequences.

Virus target	***In vitro* transcript sequence**
Jingmen tick virus	TAATACGACTCACTATAGGGAGTCACAGGAGACATTTACATCACCATCTTCAGCGCCATCACCGCTGTGGGATTATAGCGGCCGCTTATTACTAGGGCGGACGCGGCGCTATCACCGCTGTCTCCCTATGGGAATTATAGCGGCCGCTTATTATGGATGGCCGACCCCGCTATAAGGAGGATCAATGCCAATGTAAAGAACGTCAAGTCTGGATTGGTGGCTCCATCTTGGCTTCCCTGTCCACCTTCCAGCAGATGTGGATCAGCAAGCAGGAGTATGATGAGTCGCAATAACTAGCATAACCCCTTGGGGCCTCTAAACGGGTCTTGAGGGGTTTTTTGCTGA
Alongshan virus	TAATACGACTCACTATAGGGAGTCACAGGAGATCTCTACATCATCGCCTTCAGCGCCATCGCAGCTCTGGGGCCATTATAGCGGCCGCTTATTATGCTGCTAGGGCGGAAGCCGCGCTGTCTGGATTGGTGGCTCCATCTTGGCTTCCCTGTCCACCTTCCAGCAGATGTGGATCAGCAAGCAGGAGTATGATGAGTCGCAATAACTAGCATAACCCCTTGGGGCCTCTAAACGGGTCTTGAGGGGTTTTTTGCTGA
Yanggou tick virus	TAATACGACTCACTATAGGGAGTCACAGGGGACATCTACCTCATCATCTTCAGCGCCATCGCGGCTGTGGGACCATTATAGCGGCCGCTTATTATGCTGCTAGGGCGGAGTCGGCGCTGTCTGGATTGGTGGCTCCATCTTGGCTTCCCTGTCCACCTTCCAGCAGATGTGGATCAGCAAGCAGGAGTATGATGAGTCGCAATAACTAGCATAACCCCTTGGGGCCTCTAAACGGGTCTTGAGGGGTTTTTTGCTGA
Takachi virus	TAATACGACTCACTATAGGGAGTCACAGGAGACCTCTACATCATCATCTTCAGCGCAATCGCGGCGGTGGGGCCATTATAGCGGCCGCTTATTAGTCTGCAAGAGCGGACCAAGCCCTGTCTGGATTGGTGGCTCCATCTTGGCTTCCCTGTCCACCTTCCAGCAGATGTGGATCAGCAAGCAGGAGTATGATGAGTCGCAATAACTAGCATAACCCCTTGGGGCCTCTAAACGGGTCTTGAGGGGTTTTTTGCTGA
Pteropus lylei jingmenvirus	TAATACGACTCACTATAGGGAGTCACAGGAGATCTCTACATCATCGCCTTCACCGCCAACGCAGCTCTGGTGCCATTATAGCGGCCGCTTATTATTCTACTAGGGCAGAGCCCGCGCTGTCTGGATTGGTGGCTCCATCTTGGCTTCCCTGTCCACCTTCCAGCAGATGTGGATCAGCAAGCAGGAGTATGATGAGTCGCAATAACTAGCATAACCCCTTGGGGCCTCTAAACGGGTCTTGAGGGGTTTTTTGCTGA
Guangdong jingmen-like virus	TAATACGACTCACTATAGGGAGTCACGGGCGACGTTTACATCGTCACCGCAGGAGCTATTGCGGCAGTGGGACCATTATAGCGGCCGCTTATTATGCCACTAAAGCAGAAAGCATCATGTCTGGATTGGTGGCTCCATCTTGGCTTCCCTGTCCACCTTCCAGCAGATGTGGATCAGCAAGCAGGAGTATGATGAGTCGCAATAACTAGCATAACCCCTTGGGGCCTCTAAACGGGTCTTGAGGGGTTTTTTGCTGA
NotI hybridization sequence	ATTATAGCGGCCGCTTATTA
Actin sequence	GTCTGGATTGGTGGCTCCATCTTGGCTTCCCTGTCCACCTTCCAGCAGA TGTGGATCAGCAAGCAGGAGTATGATGAGTC
